# Operational momentum effect in children with and without developmental dyscalculia

**DOI:** 10.3389/fpsyg.2013.00847

**Published:** 2013-11-12

**Authors:** Karin Kucian, Fabienne Plangger, Ruth O'Gorman, Michael von Aster

**Affiliations:** ^1^Center for MR-Research, University Children's Hospital ZurichZurich, Switzerland; ^2^Children's Research Center, University Children's Hospital ZurichZurich, Switzerland; ^3^Center of Integrative Human Physiology, University of ZurichSwitzerland; ^4^Department of Child- and Adolescent PsychiatryDRK-Hospital Westend Berlin, Germany; ^5^Clinical Psychology and Psychotherapy, University of PotsdamPotsdam, Germany

**Keywords:** developmental dyscalculia, operational momentum, children, learning disability, numerical cognition, mental number line, symbolic calculation, attention

## The presence of operational momentum in childhood

In their valuable article, Knops et al. (2013) challenge the existence of the operational momentum effect (OME) in children. The OME is characterized by the tendency to overestimate the result of addition problems and to underestimate the result of subtractions (McCrink et al., 2007). In line with previous findings, they replicated the OME in adults using a non-symbolic approximation task. In contrast, children did not exhibit such an OME. This finding was quite unexpected since current studies claim that the OME is present in childhood, as early as 9 months of age (Pinhas and Fischer, 2008; McCrink and Wynn, 2009). Together with the evaluation of attentional orienting capacity, the authors concluded that an attentional shift along the mental number line (MNL) most probably explains the OME.

**Figure 1:**
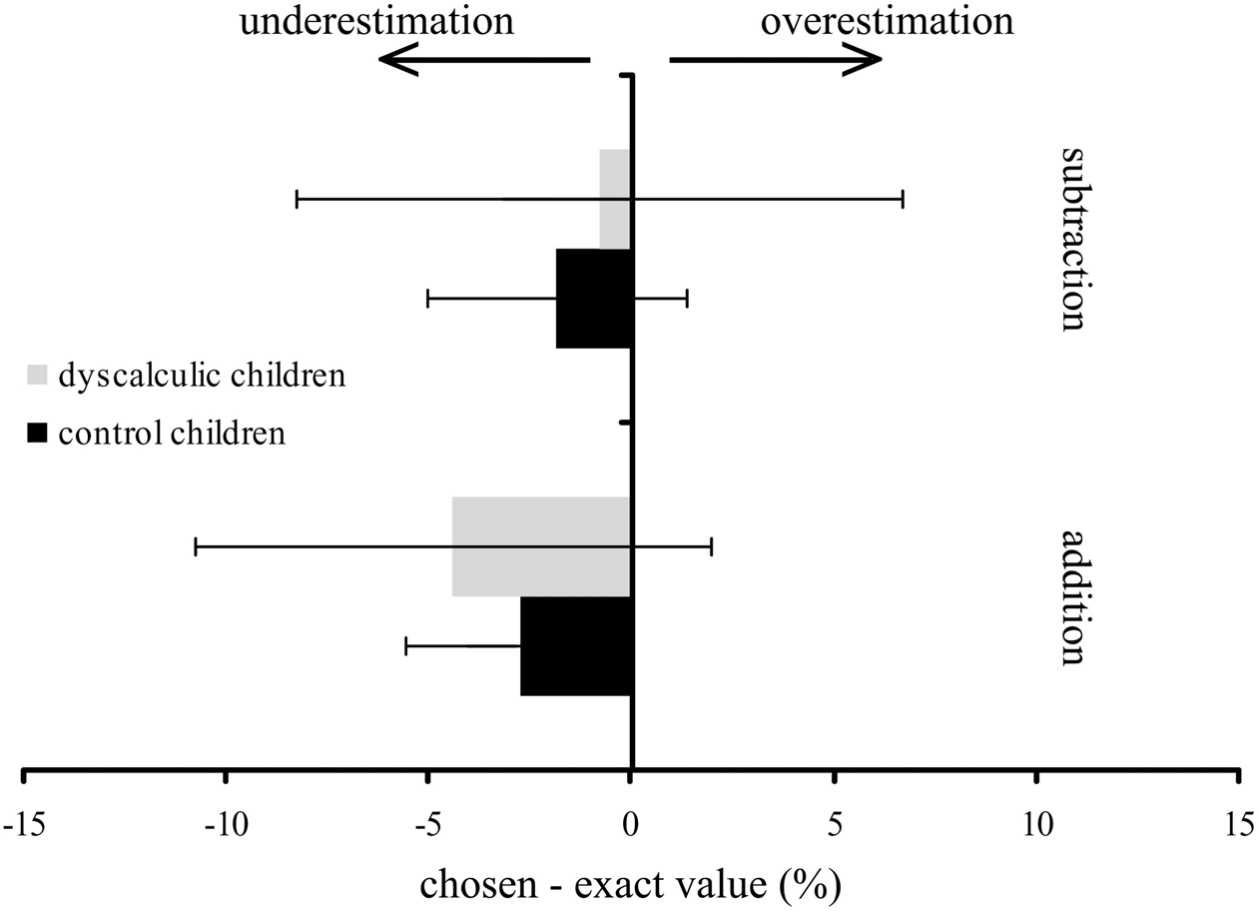
**Operational momentum effect in children with developmental dyscalculia and controls.** Mean difference between chosen and exact value on a number line from 0–100 of children with (gray) and without DD (black). Negative values indicate an underestimation and positive values an overestimation of the position on the number line.

We take the liberty of adding own results in this commentary which further support these findings. We have also tested the OME in typically achieving children and a matched group with developmental dyscalculia (DD) (Table 1). Typically achieving children did not show an OME in a symbolic number line task, as children underestimated the location of results for both additions and subtractions on the number line (Figure 1). In contrast to the study demonstrating an OME in 9-month old babies (McCrink and Wynn, 2009), we used a symbolic numerical task similar to the one used by Pinhas and Fischer (2008) who observed a reliable OME. However, their participants were already in adolescence. Therefore, it might be possible that since school-age children have lower experience in symbolic processing of calculations, an unconscious shift of attention on the MNL becomes evident only with increasing expertise and automatization.

**Table 1:** **Demographic and testmetric data**.

	**Dyscalculic children**	**Control children**	***p*-value**
N (f/m)	16 (10/6)	16 (9/7)	
Handedness (right/ambidexter/left)	14/1/1	12/0/4	
Age (years)	9.5 (0.8)	9.5 (1.1)	0.986
School grade	3.1 (1.1)	2.9 (0.9)	0.604
General IQ	99.0 (6.9)	110.1 (7.0)	0.000
Performance IQ	100.0 (12.2)	108.6 (10.2)	0.038
Verbal IQ	98.3 (6.8)	111.1 (6.7)	0.000
Numeracy (percentile rank)	14.7 (20.1)	68.9 (18.8)	0.000
Visuo-spatial memory span (block span)	4.6 (0.7)	4.9 (1.0)	0.361
Visuo-spatial working memory (block span)	2.1 (0.7)	2.8 (0.9)	0.043

General IQ was assessed by the mean of following subtests of the WISC-III (Wechsler, 1999): similarities, picture arrangement, calculation, block design, vocabulary. Performance IQ, mean of picture arrangement, calculation, block design; Verbal IQ, mean of similarities, vocabulary. Numeracy was measured by the neuropsychological test battery for number processing and calculation in children ZAREKI-R (Von Aster et al., 2006). Visuo-spatial memory span was determined by the Corsi-Block-Tapping test (Corsi, 1972) and visuo-spatial working memory by the Corsi-Block-Suppression test (Beblo et al., 2004).

Together with the findings of Knops et al., it seems that the left–right associations underlying the OME are dependent on development and experience. One might hypothesize that a complex interaction between visuo-spatial and attentional processes together with number related skills influence its development whereupon an early predisposition to relate representations of non-symbolic numerical magnitude to spatial length builds a core system (De Hevia and Spelke, 2010). In combination with cultural conventions such as reading direction, the experience of specific left-small/right-large associations (Berch et al., 1999; Opfer et al., 2010), numeric magnitude and number line estimation (Siegler and Booth, 2004) and the understanding of arithmetic concepts might lead first to the OME by non-symbolic presentation and later, after the acquisition of the symbolic number system, to the OME found by symbolic presentation (Pinhas and Fischer, 2008). That the OME is possibly subject to the dynamic nature of developing math proficiency would also be in line with current neuropsychological (Von Aster and Shalev, 2007) and neuronal models (Kucian and Kaufmann, 2009) suggesting the development of MNL representation successively depending on previous processes of representing numerical magnitudes by first verbal and later Arabic symbols. However, future studies will have to address these hypothesis systematically.

According to the assumption of such a hierarchical and interwoven model of OME, the impairment of one or several aspects may affect the development of the OME negatively.

## The OME in children with developmental dyscalculia

DD is a specific learning disability of number processing and calculation. In addition to profound problems in numerical understanding, abnormalities in visuo-spatial, and attentional processes have also been associated with DD.

As expected, no OME was observed in our tested group of children with DD (Figure 1). Therefore, one might speculate that a lack of numerical understanding and reduced visuo-spatial and attentional functions, as often found in children with DD, might hinder the development of an OME.

## Limitations

It is important to note that an adult control group is missing in our study. However, since Pinhas and Fischer (2008) demonstrated an OME in adolescents by means of a comparable paradigm to ours, we assume that the lack of OME in children is not due to differences in task design.

## Does a flawed uncompression of numerical information cause the OME?

Knops et al. argue that the lack of an OME during childhood speaks against a flawed compression-uncompression mechanism as the driving factor of the operational momentum bias, which implies that the OME is caused by a systematic bias during uncompression of a logarithmic representation of the MNL. Furthermore, number representations change from a logarithmic mapping to a linear mapping scheme during development and familiarity in a number range (Siegler and Opfer, 2003). Hence, the MNL representation cannot be assumed to be identical in children and adults examined by Knops. However, as outlined by the authors, if a flawed uncompression of numerical information causes the OME, it should be more pronounced in children who still represent numbers in a logarithmic fashion.

In contrast to the cohort of Knops et al., children in our study were older and showed a linear function describing the MNL representation like in adults, but exhibited no OME. Moreover, our results indicated that even after the completion of a specific number line training (Kucian et al., 2011), no OME was evident, although the training had a positive effect on the MNL representation. Therefore, our results further corroborate that differences in uncompression mechanisms of the MNL are unlikely to solely cause the OME.

## Conclusions

Findings reported by Knops et al. and our study corroborate that the OME is not necessarily present in childhood and unlikely to be caused by flawed uncompression of the MNL. In addition, our results further point to a possible negative impact of DD on the development of the OME. In conclusion, the OME is probably dependent on development and a complex interaction of the maturity of numerical skills, visuo-spatial, and attentional processes as well as cultural conventions.
